# The Phenotypic Radiation Resistance of CD44^+^/CD24^−or low^ Breast Cancer Cells Is Mediated through the Enhanced Activation of ATM Signaling

**DOI:** 10.1371/journal.pone.0024080

**Published:** 2011-09-15

**Authors:** Hong Yin, Jonathan Glass

**Affiliations:** Feist-Weiller Cancer Center and Department of Medicine, Louisiana State University Health Sciences Center, Shreveport, Louisiana, United States of America; Universita' di Milano, Italy

## Abstract

Cancer initiating cells (CIC) are stem-like cells. CIC may contribute not only to the initiation of cancer but also to cancer recurrence because of the resistance of CIC both to chemotherapy and radiation therapy. From the MCF-7 and MDA-MB231 breast cancer cell lines and primary culture of patient breast cancer cells, we isolated by flow cytometry a CIC subset of cells with the CD44^+^/CD24^−or low^ phenotype. The CD44^+^/CD24^−or low^ subset showed increased sphere formation and resistance to radiation compared to the non- CD44^+^/CD24^−or low^ subset. The increased radiation resistance was not dependent on the result of altered non-homologous end joining (NHEJ) DNA repair activity as both NHEJ activity and expression of the various proteins involved in NHEJ were not significantly different between the CD44^+^/CD24^−or low^ and non- CD44^+^/CD24^−or low^ subsets. However, activation of ATM signaling was significantly increased in CD44^+^/CD24^−or low^ cells compared to non- CD44^+^/CD24^−or low^ cells in both from breast cancer cell lines and primary human breast cancer cells. Application of an ATM inhibitor effectively decreased the radiation resistance of CD44^+^/CD24^−or low^ subset, suggesting that targeting ATM signaling may provide a new tool to eradicate stem-like CIC and abolish the radiation resistance of breast cancer.

## Introduction

The existence of stem-like cancer initiating cells (CIC) is a hypothesis put forth both to explain the initiation of cancer and the recurrence of cancer after treatment. Evidence supporting the presence of CIC has been developed both in hematologic malignancies and solid tumors. In breast cancer, a subset of CD44^+^/CD24^−/low^/ESA+ cells has been identified with as few as 100 cells of these cells being able to form tumors in mice [1], [2], [3]. The CD44^+^/CD24^−/low^/ESA+ cells exhibit unlimited propagation and can give rise to subpopulations of tumorigenic and non-tumorigenic cells. Therefore, the subset of CD44^+^/CD24^−/low^/ESA+ has been recognized as being breast cancer initiating cells (CIC). In breast cancers CD44^+^/CD24^−/low^ cells are predominately limited to triple negative breast cancer, a subgroup of basal-like tumors, and the presence of the CD44^+^/CD24^−/low^ subset is correlated inversely with breast cancer patient survival [4], [5]. In addition to breast cancer tissue, CD44^+^/CD24^−/low^/ESA+ cells have also been isolated from breast cancer-derived cell lines with several of such cell lines containing a subset of CD44^+^/CD24^−/low−^/EAS+ cells possessing CIC properties such as the capacity for self-renewal [1], [6], [7], [8].

One of the characteristics of CIC, including CIC isolated from breast cancer cell lines, is resistance to radiation and chemotherapy which may adversely impact cancer treatment although the mechanisms responsible for the resistance are still poorly understood [9], [10], [11], [12]. The growth of the breast cancer cell lines MCF-7 and MDA-MB-231 as mammospheres has demonstrated the enrichment in the mammospheres of CD44^+^/CD24^−/low−^/EAS+ cells and the cells in the mammospheres are more radiation resistance than cells grown in monolayer [13]. The radiation resistance of CIC has also been demonstrated in mouse mammary progenitor cells with an increase of progenitor cells with the characteristic stem cell surface markers following radiation of primary BALB/c mouse mammary epithelial cells [11]. Fractional radiation also increased the CD44^+^/CD24^−/low−^ subset in breast cancer cell lines [14]. However, due to the dynamic features of CIC, that is the need to both self-renew and to differentiate, it is unknown if the CD44/CD24 surface phenotype of CIC is directly responsible for the observed radiation resistance.

The mechanisms underlying the relative resistance of CIC to radiation and chemotherapy are important to overcoming the barriers resistance poses to more effective cancer treatment. Recent data with CIC isolated from human breast cancer cells and mouse mammary tumor cells implicate low levels of reactive oxygen species (ROS) and decreased levels of cellular defenses against oxidative stress in CIC as contributing to radiation resistance [13], [15]. In addition, enhanced DNA damage repair activity could also contribute to radiation resistance of CIC. After radiation, increased activation of Ataxia-Telangiectasia Mutated (ATM) kinase pathway has been reported in glioma stem cells andCD133-positive atypical teratoid/rhabdoid tumor cells [16], [17]. Analysis of the survival curves for radiated breast cancer cells showed a “differential shoulder region” suggestive of a difference in DNA repair between CIC and non-CIC. Therefore, targeting the differential capacity for DNA repair in CIC suggests a mechanism for obtaining enhanced therapeutic efficacy of radiation. In this study, we demonstrate that CIC isolated as a CD44^+^/CD24^−/low^/ESA+ subset of cells from both human breast cancer cell lines and primary culture of breast cells isolated from patients with fibroadenoma (patient A) and invasive ductal carcinoma (patient B) were more radioresistant than control subsets and that the radiation resistance cohort was characterized by the presence of stem cells surface markers. The radiation resistance of the human breast CIC was markedly decreased by targeting the ATM with the inhibitor KU55933.

## Results

### 1. Post-radiation survival analysis of CD44+/CD24−/or low (MCF-7, MDA-MB231, and patient's cells) vs CD44−/CD24 high (MCF-7 and patient's cells) or CD44+/CD24+ (MDA-MB-231

In previous studies, breast cancer cells cultured as mammospheres in serum-free medium are recognized as a CIC enriched subset and show enhanced radiation resistance and increased percentage of CD44^+^/CD24^−or low^ cells [13], [18]. According to the previous studies, we assume that the CD44^+^/CD24^−or low^ cells could be CIC enriched subset in our study and this subset is directly responsible for enhanced radiation resistance. To test this assumption, we isolated CD326(ESA)^+^/CD44^+^/CD24^−or low^ cells not only from the breast cancer cell lines MCF7 and MDA-MB231 but also from primary cultures of human breast tumor cells. CD326 positive cells constituted 99% of MCF7 cells, 70% of MDA-MB231 cells, 73% of primary cells from patient A, and 95% of cells from patient B (Figure S1A–D, left panels). The CD326 subset was then examined for the presence of CD44 and CD24. The CD44^+^/CD24^−or low^ subset constituted 0.1% of the MCF-7 cells and 86% of the MDA-MB-231 cells (Figure S1 A and S1 B, right panels). In patients A and B, the CD44^+^/CD24^−or low^ subset of cells was 7.4% and 0.1%, respectively (Figure S1 C and S1 D, right panels). The greatest portion of a CD44^+^/CD24^−^ subset was found in the mesenchyme-like breast cancer cell line MDA-MB-231 as described before [8], [19], [20] and this cell line has previously been characterized as having a basal cell phenotype [21].

**Figure 1:**
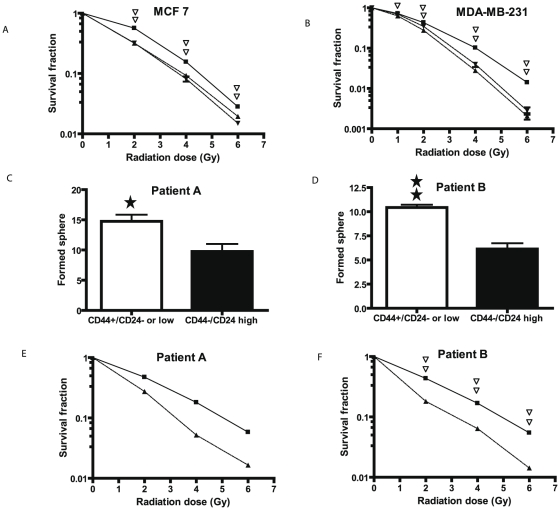
Post-radiation clonogenic survival and mammosphere formation analyses of the CD44^+^/CD24^−or low^ subset vs. CD44^−^/CD24^high^ (MCF-7, Patient A, and Patient B) and CD44^+^/CD24^+^ (MDA-MB-231) subsets. A and B. As detailed in the Methods radiation dose-response curves were performed either of unsorted MCF-7 cells (▾) or the MCF-7 cell subsets CD44^+^/CD24^−or low^ (▪) and CD44^−^/CD24^high^ (▴) (Panel A) or unsorted MDA-MB-231 cells (▾), or the MDA-MB-231 cell subsets CD44^+^/CD24^−^ (▪) or CD44^+^/CD24^+^ (▴) (Panel B). Double open triangles indicate a significant difference at the indicated dose of radiation with a P<0.01 between the CD44^+^/CD24^−or low^ subset and the CD44^−^/CD24^high^ subset or unsorted cells for MCF-7 cells and between the CD44^+^/CD24^−^ subset and the CD44^+^/CD24^+^ subset or unsorted cells for MDA-MB-231 cells. C. and D. Cells were cultured to form mammospheres from the CD44^+^/CD24^−or low^ or the CD44^+/^CD24^high^ subsets of Patient A (Panel C) and Patient B (Panel D) as detailed in the Methods. In brief, the cells sorted from primary cultures were grown on low cell binding 96 well plates at a density of 200 cells in 0.2 ml of medium per well. The spheres formed were counted at day 12. The * indicates a significant difference with P<0.05 and ** indicates a significant difference with P<0.01 between the CD44^+^/CD24^−or low^ subset and CD44^−^/CD24^high^subset. E and F. A radiation dose response curve was performed on the CD44^+^/CD24^−or low^ subset (▪) and the CD44^+^/CD24^+^ subset (▴) from Patient A (Panel E) and Patient B (Panel F) as detailed in the Methods. Double open triangles indicate a significant difference at the indicated radiation dose with P<0.01 between the CD44^+^/CD24^−or low^ subset and the CD44^−^/CD24^high^ subset in patient B cells.

We first examined radiation sensitivity of the various CD44/CD24 subsets in MCF and MDA-MB-231 cells by assaying post-radiation clonogenic survival. A significant resistance to radiation (P<0.01) was seen in the CD44^+^/CD24^−or low^ subsets of cells from MCF-7and MDA-MB-231 cells compared to that of CD44^+^/CD24^high^ subset in MDA-MB-231, CD44^−^/CD24^high^ subset in MCF-7 or unsorted cell populations (Figure 1A and 1B). To verify results from the breast cancer cell lines, we performed similar studies on isolated CD44^+^/CD24^−or low^ subsets from primary culture of benign breast tumor (patient A) and an invasive breast cancer cells (patient B). First, we examined the stem-like property of isolated cells by sphere formation assay. By 12 days after the culture in serum-free medium, the CD44^+^/CD24^−or low^ subset formed 62 and 69% more spheres than did CD44^−^/CD24^high^ subsets in patient A and B, respectively (Figure 1 C and 1D, P<0.05). Radiation survival curves showed that the CD44^+^/CD24^−or low^ subsets from both patients were more resistant compared to the CD44^−^/CD24^high^ subsets (Figure 1E and 1F). At 2Gy the survival fractions of the CD44^+^/CD24^−or low^ subset vs CD44^−^/CD24^high^ subset was 0.47 vs 0.27 in patient A (P<0.01) and 0.43 vs 0.18 in patient B (P<0.01).

### 2. The radiation resistance of breast cancer cells is related to CD44^+^/CD24^−or low^ phenotype

**Figure 2:**
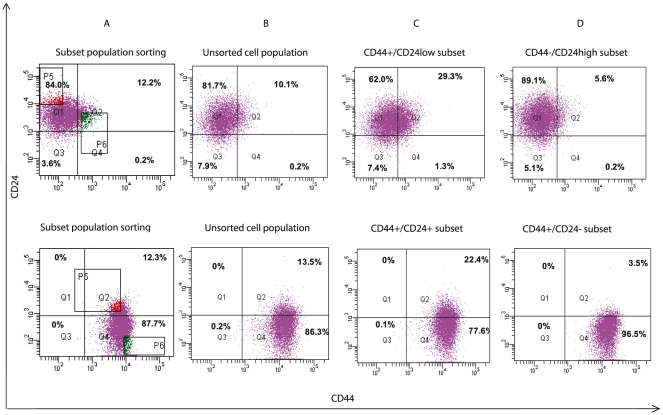
CD44/CD24 expression after culture of sorted cells. MCF-7 (top panel) and MDA-MB-231 (bottom panel) cells were sorted by the surface markers CD44 and CD24 as shown (column A) into CD44^+^/CD24^−or low^ (3.0%) and CD44^−^/CD24^high^ (3.2%) subsets for MCF-7 or CD44^+^/CD24^−^ (4.5%) and CD44^+^/CD24^+^ (4.5%) for MDA-MB-231. The parent cell lines (column B) and the subsets (columns C and D) were cultured for 20 days and re-examined for CD44 and CD24 expression. The fluorescent intensity of CD 24 expression is on the Y axis and of CD44 expression on the X axis. Shown is the percentage of cells in each quadrant.

The stem-like phenotype of CIC is a dynamic process in that the CIC must undergo continued “differentiation” to form the bulk of the cancer and self-renewal to maintain the capacity for continued stemness or stem-like phenotype. We next examined if the sorted CIC subset maintained their surface phenotype during subsequent culture. In these experiments CD44/CD24 expression was examined in the MCF-7 and MDA-MB-231 cells, sorting these cell lines into CD44^+^/CD24^−/low^ and CD44^−^/CD24^high^ subsets for the MCF-7 cell line and CD44^+^/CD24^−^ and CD44^+^/CD24^+^ subsets for the MDA-MB-231 cell line. After 7 days of culture both the MCF7 and MDA-MB-231 subsets as well as the unsorted parent cells retained their original CD44/CD24 phenotypes (data not shown). However, after 20 days of culture the CD44^+^/CD24^−/low^ MCF7 subset had changed phenotype with 69.4% of the cells now CD44^−^ and in the CD44^−^/CD24^high^ subset 5.8% of the cells were now CD44^+^ (Figure 2, upper panel). In the MDA-MB-231 cells similar phenotype changes were also observed after 20 days (Figure 2, lower panel) with 77.7% of the cells in the CD44+/CD24+ subset becoming CD24− and 3.5% of CD24− cells in CD44+/CD24− subset becoming CD24+. It seems that the stem-like cell phenotype could switch to a non stem-like cell phenotype and vice versa.

**Figure 3:**
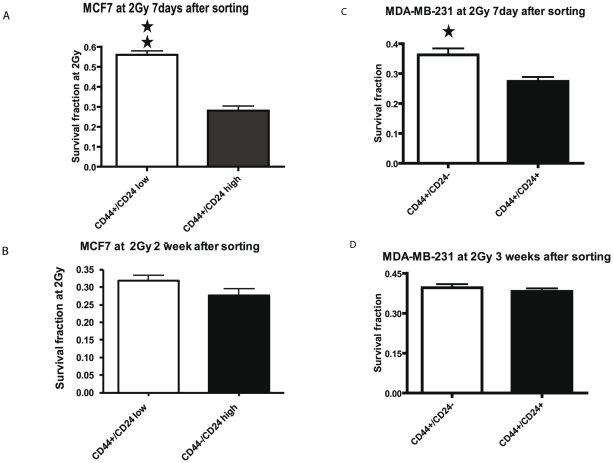
The affect of culture on the radiation resistance of stem-like subsets. MCF-7 cells (Panels A and B) were sorted into CD44^+^/CD24^low^ (open bars) and CD44^−^/CD24^high^ (solid bars) subsets. MDA-MB-231 cells (Panels C and D) were sorted into CD44^+^/CD24^−^ (open bars) and CD44^+^/CD24^+^ (solid bars) subsets. A clonogenic survival analysis was conducted with calculation of the survival fraction at 2 Gy of radiation after 7 days (A and C), two weeks (B), or three weeks (D) of culture. Each column represents the mean of three independent experiments ± SD. The single and double stars represent a significant difference between two subsets in MCF7 and MDA-MB-231 cells with P<0.05 and P<0.01, respectively.

**Figure 4:**
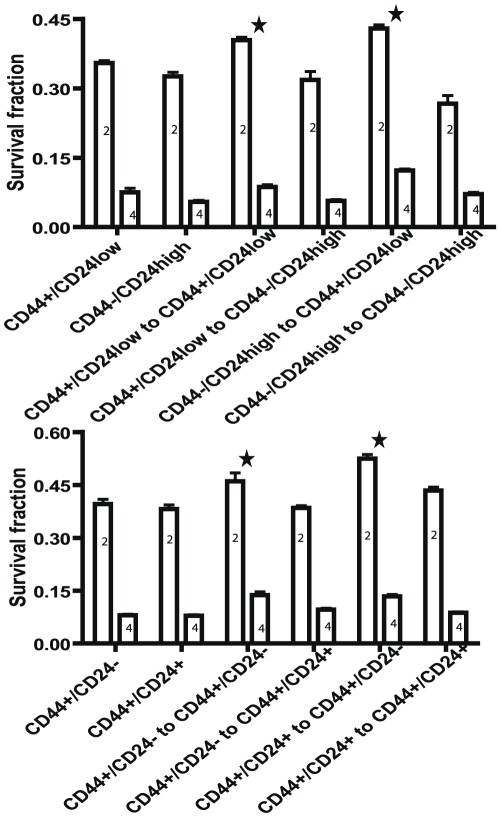
Analysis of radiation resistance of stem-like subsets upon initial isolation and on the same subset obtained upon resorting after culture. MCF7 cells (upper panel) were sorted into CD44^+^/CD24^low^ and CD44^−^/CD24^high^ subsets and MDA-MB-231 cells (lower panel) were sorted into CD44^+^/CD 24^−^ and CD44^+^/CD 24^+^ subsets. All four subsets were cultured for 20 days, resorted into the same subsets and clonogenic survival analyses were performed after radiation with either 2 Gy (2) or 4 Gy (4). Shown are the means ± SD of 3 experiments. Statistical analyses were performed with two-way ANOVA analysis. The stars indicate a significant difference with P<0.01 in the survival fraction of the resorted CIC group compared to the resorted non CIC groups. In upper panel CD44^+^/CD24^low^ and CD44^−^/CD24^high^ mean 20 day cultured MCF7 subsets. CD44^+^/CD24^low^ to CD44^+^/CD24^low^ and CD44^+^/CD24^low^ to CD44^−^/CD24^high^ indicate that cells were re-sorted into CD44^+^/CD24^low^ and CD44^−^/CD24^high^ subsets from 20 day cultured CD44^+^/CD24^low^ subset. CD44^−^/CD24^high^ to CD44^+^/CD24^low^and CD44^−^/CD24^high^ to CD44^−^/CD24^high^ indicate that cells were re-sorted into CD44^+^/CD24^low^ and CD44^−^/CD24^high^ subsets from 20 day cultured CD44^−^/CD24^high^ subset. In lower panel CD44^+^/CD24^−^ and CD44^+^/CD24^+^ mean 20 day cultured MDA-MB-231 subsets. CD44^+^/CD24^−^ to CD44^+^/CD24^−^ and CD44^+^/CD24^−^ to CD44^+^/CD24^+^ indicate that cells were re-sorted into CD44^+^/CD24^−^ and CD44^+^/CD24^+^ subsets from 20 day cultured CD44^+^/CD24^−^ subset. CD44^+^/CD24^+^ to CD44^+^/CD24^−^and CD44^+^/CD24^+^ to CD44^+^/CD24^+^ indicate that cells were re-sorted into CD44^+^/CD24^−^ and CD44^+^/CD24^+^ subsets from 20 day cultured CD44^+^/CD24^+^ subset.

To verify further a relationship between the CD44^+^/CD24^−or low^ phenotype and radiation resistance, we compared the clonogenic survival of MCF-7 and MDA-MB-231 subsets to radiation after culture for 7 days and 2 weeks for MCF-7 or 3 weeks for MDA-MB-231. These experiments took advantage of the surface phenotype of the sorted cell subsets being maintained for 7 days but not 20 days of culture. Indeed, the clonogenic survival fraction at 2Gy of radiation was significantly greater in the seven day-culture of the MCF7 CD44^+^/CD24^−or low^ subset compared to that of CD44^−^/CD24^high^ subset (Figure 3A) but by two weeks of culture the radiation resistance of the CD44^+^/CD24^−or low^ subset was no longer significantly different than the CD44^−^/CD24^hgh^ subset (Figure 3B). Similarly, with the MDA-MB-231 subsets the increased survival fraction of the CD44^+^/CD24^−^ subset compared to the CD44^+^/CD24^+^ subset after 7 days of culture disappeared in the 20-day cultures (Figure 3C and 3D). We then resorted the MCF7 and MDA-MB-231 subsets after 20 days in culture into CIC and non CIC subsets and examined by clonogenic assays the radiation survival among the 20 day cultured subsets and re-sorted subsets (Figure 4). In both the MCF7 and MDA-MB-231 cell lines, the resorted CIC subsets are significantly radiation resistant (P<0.01) whether sorted from the original CIC subsets or not, indicating that CD44^+^/CD24^−/low^ phenotype determine the radiation resistance of the cells.

### 3. Analysis of end-joining of double-strand DNA breaks in CD44^+/^CD24^−or low^ vs. CD44^−^/CD24^high^ (MCF-7 and HCC1937) or CD44+/CD24+ (MDA-MB-231) subpopulations

DNA repair via non-homologous end joining (NHEJ) is one of the immediate responses of cells to radiation-induced DNA damage. To address if the enhanced radiation resistance of CD44^+^/CD24^−or low^ subsets was due to increased DNA repair activity, we first examined in the MCF-7, HCC1937, and MDA-MB-231cell lines NHEJ activity *in vivo* using a linearized plasmid containing the luciferase reporter in the putative CIC containing populations compared to the appropriate non CIC subsets. As shown in Figure S2A, no increase of *in vivo* NHEJ was seen in MCF-7 cells with the CD44^+^/CD24^−or low^ phenotype compared to the CD44^−^/CD24^+^. In contrast, increased *in vivo* NHEJ was found in the CD44^−^/CD24^high^ subset of HCC1937 and the CD44^+^/CD24^+^ subset of MDA-MB-231 cells. However, the increased *in vivo* NHEJ may merely reflect the low transfection efficiency in the CD44^+^/CD24^−^ subsets of both cell lines as low expression of the co-transfected reference gene, β-galactosidase, was always noted. Further analysis of NHEJ function *in vitro* with MDA-MB-231 cells showed that there was not a significant difference in DNA ligation between CD44^+^/CD24^−^ and CD44^+^/CD24^+^ subsets of MDA-MB-231 cells (Figure S2B). Combined with post-radiation clonogenic survival results, the NHEJ analyses suggest that enhanced clonogenic survival in CD44^+^/CD24^−or low^ subsets may not be a function of altered NHEJ activity.

### 4. Protein expression of DNA damage/repair response genes after radiation in CD44^+^/CD24^−or low^ subpopulations

**Figure 5:**
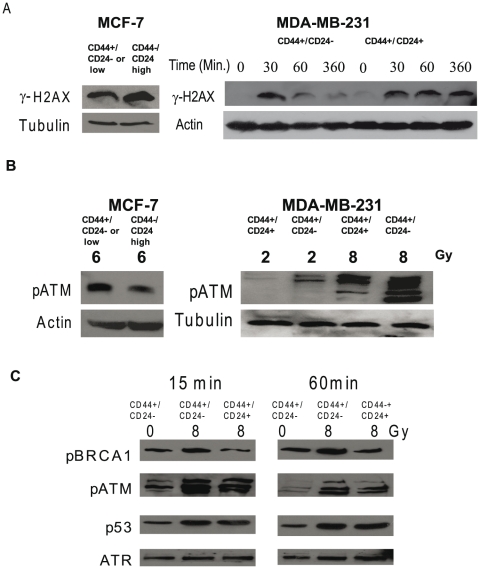
The differential response to radiation correlates with differential phosphorylation of ATM and its downstream targets between CD44^+^/CD24^−^ and CD44^+^/CD24^+^ subsets in MDA-MB231 cells and between CD44^+^/CD24^low^ and CD44^−^/CD24^high^ subsets in MCF-7 cells. The indicated sorted subsets of MCF-7 and MDA-MB231 cells were cultured for 5 days as described in the Methods and radiated with the indicated doses. Whole cell extracts (MCF-7) or nuclear extracts (MDA-MB-231) were analyzed by western blot analysis for the presence of phosphorylated γH2AX (**A**), phosphorylated ATM (**B**), and BRCA1 and p53 (**C**) the downstream targets of phosphorylated ATM. between CD44^+^/CD24^−^ and CD44^+^/CD24^+^ subsets of MDA-MB231 cells. In **C** western blot analyses were conducted for time-related changes in phosphorylated ATM, phosphorylated BRCA1, and phosphorylated p53. The ATR expression was utilized as a loading control.

To further investigate the mechanisms of enhanced radiation resistance in CD44^+^/CD24^−or low^ subsets, we examined the expression of DNA damage/repair related proteins before and after radiation by western blot analysis (Figure S3 and Figure 5). We first examined the expression of proteins responsible for NHEJ and showed that Ku70, Ku 80, PARP, and DNA-PKcs were not differentially expressed between CD44^+^/CD24^−^ and CD44^+^/CD24^+^ MDA-MB-231 cells (Figure S3). These results supported our observation that NHEJ did not contribute to enhanced radiation resistance in the CD44^+^/CD24^−^ cell subset. However, the investigation of radiation-mediated DNA damage disclosed that phosphorylated histone 2AX (γ-H2AX), a marker of double stranded DNA damage, was decreased by half in CD44^+^/CD24^−or low^ MCF-7 cells and ∼three fold in CD44^+^/CD24^−^ MDA-MB-231 cells compared to the corresponding control subsets one hour after radiation (Figure 5A). In the MDA-MB-231 subset of CD44^+^/CD24^−^ cells, γ-H2AX underwent faster dephosphorylation than the CD24^+^ subset with γ-H2AX expression reduced by 75% in the CD44^+^/CD24^−^ subset 6 hours after radiation while in CD44^+^/CD24^+^ subset expression of γ-H2AX did not change over time, suggesting delayed DNA repair in CD44^+^/CD24^+^ cells.

H2AX is known to be phosphorylated by ATM and other PIKK kinases [22]. In response to DNA damage, ATM is first activated through auto-phosphorylation and by other kinases [23]. Hence, it was of interest to determine if ATM activation was differentially affected in the CIC and non-CIC subsets following radiation. In fact, one hour after radiation ATM phosphorylation in the CD44^+^/CD24^−or low^ subsets was significantly increased by 27% in MCF-7 cells compared to the corresponding control subsets (Figure 5B, left panel). At 2 Gy, a level similar to a clinical dose of radiation, the CD44^+^/CD24^−^ subset of MDA-MB-231 cells showed a seven fold increase of ATM phosphorylation compared to the CD44^+^/CD24^+^subset (Figure 5B, right panel). Interestingly, the ATM partner, ATR, was not differentially phosphorylated in the two subsets before and after radiation (Figure S4A). Expression of two other components of the DNA damage-recognizing complex, MRE 11 and Nibrin, was similar between the two subsets of MDA-MB-231 cells (Figure S4B). The phosphorylation of ATM in the CD44^+^/CD24^−^ subset was rapidly affected by radiation increasing by 85% 15 minutes after radiation and was still increased by 40% after 60 minutes with far smaller changes seen in the CD44^+^/CD24^+^ subset (Figure 5C). Phosphorylation of the ATM target, BRCA1 also was slightly increased by ∼1.3 and 1.5 fold in the CD44^+^/CD24^−^ subset at 15 and 60 minutes compared to the CD44^+^/CD24^+^ subset (Figure 5C) while there was no differential affect on p53 phosphorylation. In addition, we did not detect the expression of phosphorylated checkpoint proteins, Chk1 and Chk2, under our experimental conditions.

### 5. ATM inhibitor decreased the survival of the CD44+/CD24− subpopulation after radiation

**Figure 6:**
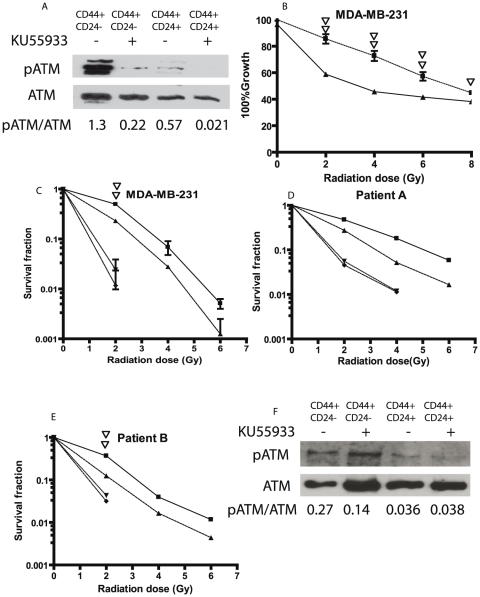
The ATM inhibitor KU55933 decreases the radiation resistance of stem-like MDA-MB-231, Patient A, and Patient B cells. Cells sorted and cultured as in Figure 1 were treated with ATM inhibitor KU55933. **A**. Western blot analysis of phosphorylated ATM 30 minutes after radiation at 10Gy in MDA-MB-231 cells treated with 20 µM of the ATM inhibitor KU55933 for two hours before radiation. **B**. The proliferation analysis of ATM inhibitor treated cells. MDA-MB-231 cells were treated with the 20 µM of KU55933 from two hours before radiation to 24 hour after radiation and cell numbers enumerated as described in the Methods after 7 days in culture. The results are expressed as the percent of cells compared to the non-radiated control and represent the mean ± SD of three independent experiments. The solid squares represent the CD44^+^/CD24^−^ subset, the triangles, the CD44^+^/CD24^+^ subset. Single and double open triangles indicate a significant difference with P<0.05 and 0.01 between KU55933 treated and untreated CD44^+^/CD24^−^ subset of MDA-MB-231 cells. **C**, **D**, and **E**. The clonogenic survival analysis of the CD44^+^/CD24^−^ subset vs. CD44^+^/CD24^+^ subset in MDA-MB-231 cells (C) and CD44^+^/CD24^−or low^ subset vs CD44^−^/CD24^high^ subset in Patient A (D) and Patient B cells (E). Sorted cells were treated with 10 µM of KU55933 one hour before the radiation and then irradiated as described in Materials and Methods. 48 hour after radiation, medium was replaced with fresh medium without KU55933. The colonies were counted at day 12. The solid square represents the CD44^+^/CD24^−^ subset in C and CD44^+^/CD24^−or low^ subset in D and E. The ▴ triangle represents the CD44^+^/CD24^+^ subset in C and the CD44^−^/CD24^high^ subset in D and E. The ▾ triangle represents the KU55933 treated CD44^+^/CD24^−^ subset in C and CD44^+^/CD24^−or low^ subset in D and E. The ♦ represents KU55933 treated CD44^+^/CD24^+^ subset in C and CD44^−^/CD24^high^ subset in D and E. Double open triangles indicate a significant difference at 2Gy with P<0.01 between KU55933 treated and untreated CD44^+^/CD24^−^ subsets in C and between the KU55933 treated and untreated CD44^+^/CD24^−or low^ subset in E. F. Western blot analysis of phosphorylated ATM 60 minutes after radiation at 6 Gy in Patient B cells treated with 10 µM of the ATM inhibitor KU55933 for one hour prior to radiation.

**Figure 7:**
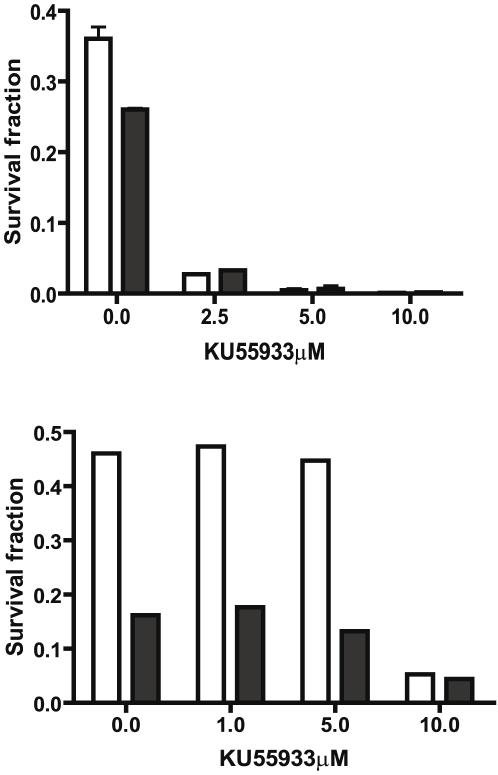
Dose-effect relationship of KU55933 and clonogenic survival fraction in MDA-MB-231 and Patient B cells. Sorted MDA-MB-231 (A) and patient B (B) cells were treated with various concentrations of KU55933 as indicated for one hour before the radiation and then irradiated at 2Gy. The colonies were enumerated as in Figure 6 and the survival fractions were calculated The open bar represents the CD44^+^/CD24^−^ subset in A and the CD44^+^/CD24^−or low^ subset in B. The solid bar represents the CD44^+^/CD24^+^ subset in A and CD44^−^/CD24^high^ subset in B.

In view of the increased phosphorylation of ATM in the CD44^+^/CD24^−^ subset in response to radiation and the important function of activated ATM in the DNA repair process, we hypothesized that differential activation of ATM was responsible for increased radiation resistance in the CD44^+^/CD24^−^ subset. To test this hypothesis, subsets of MDA-MB-231cells were treated with KU55933 (2-Morpholin-4-yl-6-thianthren-1-yl-pyran-4-one), an ATM kinase inhibitor, starting 2 hours before the radiation. Western blot analysis showed a significant inhibition of ATM phosphorylation 30 minutes after 10 Gy of radiation (Figure 6A). The proliferation assay performed 7 days after radiation showed that in the CD44^+^/CD24^−^ subset, cells treated with KU55933 were more sensitive to radiation than non-treated cells (Figure 6B). The differential response of cells to radiation between the treated and non-treated groups was especially significant at the lower radiation doses. At 2Gy and 4Gy, the growth of cells treated with the ATM inhibitor was reduced by 32% and 37%, respectively, compared to growth of the non-treated cells. The analysis of clonogenic survival showed that KU55933 decreased the survival fraction by ten-fold in both CD44^+^/CD24^−^ and CD44^+^/CD24^+^ MDA-MB231 subsets (Figure 6C). Notably, treatment with KU55933 attenuated the relative radiation resistance of CD44^+^/CD24^−^subset compared to the CD44^+^/CD24^+^ subset with the survival fractions after treatment with KU55933 being similar for both subsets with no colonies detected for radiation greater than 2 Gy. In addition to the breast cancer cell line, KU55933 had a similar effect on the two primary cultures of breast tumor, patients A and B (Figure 6D and 6E). The western blot analyses of phosphorylated ATM after radiation at 6Gy showed that the ratio of phosphorylated ATM to total ATM which was 0.27 and 0.14 in the untreated CD44^+^/CD24^−/low^ and CD44^−^/CD24^high^ subsets, respectively, decreased to 0.04 and 0.04 in the KU55933 treated CD44^+^/CD24^−^ and CD44^−^/CD24^high^ subsets in patient B. In a limited KU55933 dose response curve both the CD44^+^/CD24^−^ and CD44^+^/CD24^+^ subsets from the MDA-MB231 cells were exquisitely sensitive with the survival fractions after 2 Gy decreased by 12 fold by 2.5 µM KU55933 (Figure 7A). However, the subsets from patient B cells were less sensitive with radiation resistance being attenuated only at 10 µM KU55933 (Figure 7B). These results suggest that the activation of ATM signaling could be important for stem-like CIC survival and/or recovery post-radiation. Inhibition of ATM could be an effective treatment to attenuate the radiation resistance of the CIC of breast cancer.

## Discussion

The presence of several surface markers has been utilized for the isolation of stem-like CIC in breast cancer cell lines and tumor tissue cells. Most notably, a subset of CD44^+^/CD24^−/low^ cells isolated from breast cancers appear enriched in CIC because of the high potential tumorigenicity of these subsets. Other methods to isolate CIC such as dye exclusion mediated by the membrane transporter, ABCG2, or high expression of aldehyde dehydrogenase (ALDH) also appear to gate a subset of breast tumor cells with the capacity to initiate tumors in a mouse model [24], [25], [26]. However, the CIC enriched cell subsets isolated by different markers do not always overlap. For example, in our studies the CD44^+^/CD24^−^ cells isolated from the MCF-7 breast cancer cell line consisted of only 0.1% of the analyzed cells while the subset of Hoechst 33342 excluding cells constituted 1.3% of the same population.(data not shown) Likewise, in MDA-MB-231 cells, CD44^+^/CD24^−^ cells were >85% of the cell population while Hoechst 33342 excluding cells were less than 0.1% (data not shown). The HCC38 cell line had neither cells with the CD44+/CD24− phenotype nor any cells with the capacity to exclude the Hoechst 33342 dye but 3.9% of cells expressed ALDH (data not shown). Hence, while the CIC enriched subset may be isolated from breast cancer cell lines by the CD markers CD44 and CD24, ALDH, or membrane labeling dye PKH26 [14], [24], [27], obtaining pure cancer stem cell subsets remains a challenge in methodology. In the current studies, we utilized CD236/CD44/CD24 for isolation of CIC of breast cancer cells based on previous studies showing that ESA+/CD44^+^/CD24^−or low^ breast cancer cell subset from human breast cancer tissue and breast cancer cell lines is more tumorigenic and more stem-like behavior. In addition, CD44/CD24 has been applied as a criteria marker for determining the stem-like CIC among breast cancer cell lines treated with various factors to favor the growth of or transition to stem-like cells. For example, mammosphere culture is used as a method to verify the existence of stem-like cells with the stemness of the resulting spheres being evaluated by CD44/CD24 expression [13]. Additionally, following a low dose of radiation an increase in stem-like cells in the irradiated breast cancer cell population was detected by flow cytometric analysis utilizing the CD44/CD24 criteria for CIC [14]. The stemness of MCF-7 cells with an active OCT4 promoter was also determined with CD44/CD24 [28]. CD24^−^/ESA^+^ cells isolated from the MDA-MB-231 cell line showed accelerated growth of tumors in a xenograft model and were also more radiation resistant [29]. With these examples as precedent we assumed that selection of ESA^+^/CD44^+^/CD24^−^ cells from breast cancer cell lines and from patient breast tissue would be enriched in stem-like CIC. Our enhanced mammosphere formation with patient breast cancer cells provided further support for our assumption. In addition, clonogenic survival analysis indicated that the populations enriched in CIC cells are more radiation resistant than are the corresponding control subsets, even in monolayer culture. The radiation resistance of the CD44^+^/CD24^−/low^ subset was not only exhibited in breast cancer cell lines and primary culture of breast cancer cells but also in benign tumor cells.

We were interested if the sorted subsets would maintain their phenotype over time in culture or revert back to the pre-sort equilibrium. Within the limits of two weeks of observation the surface markers reverted back toward the presort pattern. That is, the isolated CIC characterized by the CD44^+^/CD24^−/low^ surface phenotype proliferated in culture and after 14 days developed a non-CIC phenotype. It was anticipated that the CIC would revert back to the equilibrium condition that prevailed prior to the sort. It is interesting, however, that the cells in the non-CIC subset could not only renew themselves but also on subsequent sorts could be seen to contain some cells with the stem-like CD44^+^/CD24^−^ phenotype. This phenomenon is not caused by accidently contamination. Because two sorting gates were not overlapped and post-sorting demonstrated no contamination with each other. Therefore, our observations challenge the stemness of isolated stem-like CIC by raising two questions: 1) do the purported CIC subsets really represent stem cells, and 2) does reversal of differentiation exist in cancer cells? These two questions actually bring us back to the long-lasting debate about the origin of cancer and the stem cell versus de-differentiation theories [30]. The first question is hotly debated but as CIC's have tumorigenic potential and therapeutic resistance while the non-CIC do not [24], [31], [32], [33], stem-like CIC's seem to exist. As for the second question, the mechanisms for the reversal of differentiation are not currently understood. In the larger sense, cancer cells often exhibit reversed differentiation expressing proteins usually expressed in the embryonic phase of normal tissue development. In addition, genetic instability of cancer cells could provide a mechanism for reversal of differentiation. The phenomenon of reversal of differentiation was not only exhibited with the altered expression of CD44/CD24 surface markers during culture but was also noticed in altered expression of ABCG2 in breast cancer cells [34]. Most importantly, in our studies as an example of reversal of differentiation, is that radiation resistance appears to be directly related to the surface phenotype and after two or three weeks of culture with the CD44^+^/CD24^−^ phenotype reverting to the CD44^−^/CD24^+^ phenotype radiation sensitivity was restored (Figure 3). A recent report indicates that epithelial cells can generate cells with properties of stem cells through epithelial-mesenchymal transition [35] suggesting that non stem-like cancer cells may become stem-like cells through de-differentiation or reprogramming and through the epithelial-mesenchymal transition.

The reasons for enhanced radiation resistance of CIC are not completely understood. The therapeutic effect of radiation on cancer cells is partially mediated by free radicals and hence by the production of reactive oxygen species (ROS). The basal ROS level is lower in CIC than that in non-CIC and the CIC also have enhanced antioxidant defenses with maneuvers which increased ROS levels also restoring radiation sensitivity [15]. In addition to the balance between ROS production and antioxidant systems, other factors that may contribute to CIC radiation resistance include the ability to repair radiation-mediated cell damage.[18] The repair of radiation induced double strand DNA breaks is conducted through non-homologous end joining (NHEJ) and homologous recombination (HR). We found no differences in the NHEJ activity in the CD44^+^/CD24^−^ and non CD44^+^/CD24^−^ subsets nor were there any significant differences following radiation. While we did not directly measure the HR activity in the CD44^+^/CD24^−^ and non CD44^+^/CD24^−^ subsets we did examine the activation of Ataxia-Telangiectasia Mutated Kinase (ATM), which is an initiating factor for HR, and found that the ATM activity was increased in the CD44^+^/CD24^−^ subset as was BRCA1 which is a down-stream target of ATM. The enhanced activation of ATM in CD44^+^/CD24^−^ subset was manifest both as increased phosphorylation of ATM and also as delayed dephosphorylation. In contrast, phosphorylated γ-H2AX, a marker of DNA double strand breaks and a protein which is phosphorylated by ATM, was not increased in CD44^+^/CD24^−^ subset compared to non CD44^+^/CD24^−^ subset and the phosphorylated levels of γ-H2AX subsided more quickly in the CD44^+^/CD24^−^ subset than the non CD44^+^/CD24^−^ subset. Perhaps, in CIC the persistence of phosphorylated ATM and the rapid decrease of phosphorylated γ-H2AX allow for more rapid DNA repair and hence enhanced cell survival.

ATM is a Ser/Thr kinase and belongs to phosphatidylinositol 3′ kinase-related kinase family. The functions of ATM have been well reviewed [36], [37]. ATM participates in multiple stress-related activities and plays a critical role in the maintenance of genome stability and integrity with mutations of ATM closely related to cancer predisposition including breast cancer. In response to double strand DNA damage, ATM is activated through auto-phosphorylation at several residues including Ser1981 and by poly(ADP-ribosyl)ation by PARP protein [38]. Activated ATM can initiate homologous recombination repair by phosphorylating a series of substrate proteins. Activated downstream targets of ATM/ATR after radiation may consist of over 900 proteins [39].The In glioblastomas and atypical teratoid/rhabdoid tumors, stem cells characterized by the CD133^+^ phenotype demonstrated increased radiation resistance accompanied by enhanced expression of phosphorylated ATM and the checkpoint proteins Chk1, Chk2, and Rad17 compared to CD133^−^ cells [16], [17]. The checkpoint kinase inhibitor debromohymenialdisine (DBH) effectively eliminated the radiation resistance of the CD133^+^ cells [32]. In our studies both the breast cancer cell lines MDA-M-231 and MCF-7 and the primary culture of patient breast cancer cells demonstrated enhanced expression of phosphorylated ATM in the CD44^+^/CD24^−or low^ subpopulation after radiation. In contrast to the findings with CD133^+^ positive cells from brain tumors, only slightly increased activation of downstream pathways was found primarily in BRCA1 although additional downstream targets of ATM in breast cancer CIC need to be examined.

Recently, ATM inhibitors were demonstrated to enhance radiation sensitivity of cancer cells and suppression of the proliferation of tumor cells [40], [41], [42]. In an initial study we used the ATM inhibitor KU-55933 [43] and showed significantly decreased radiation resistance of the CD44^+^/CD24^−^ subset isolated both from of the MDA-MB-231 cell line and the primary culture of patient breast cancer cells. These results suggest that ATM signaling could be an important target for radiation resistance of CIC and development of new therapeutic strategies aimed at ATM may provide a way to extinguish CIC in breast cancer.

## Materials and Methods

### Cell culture, antibodies, and reagents

The breast cancer cells lines, MCF7and MDA-MB-231, MDA-MB-436, BD20, HCC38, and HCC1937 were purchased from ATCC and cultured according to the ATCC instructions. The primary breast cells were collected from patients under the exempted protocol (Protocol Number: E09-032) approved by the Institutional Review Board for Human Research Subjects of Louisiana State University Health Sciences Center-Shreveport. No patient can be identified. Briefly, the breast tissues were disassociated with collagenase I (Invitrogen, Carlsbad, CA) at 200 unit/ml at 37C° overnight. After centrifugation and washing, the isolated small organoids and cells were cultured on Primaria Plates (Becton Dickinson, Franklin Lakes, NJ) in WIT medium (Stemgent, Cambridge, MA). In some cases, the medium was supplemented with 2% FBS to promote the growth of breast epithelial cells. The isolated breast cells were used within five to six passages. Antibodies (with catalogue numbers in parentheses) for use in western blot analysis were obtained from: 1) Cell Signaling Technology, Inc. (Danvers, MA) against phosphorylated ATM (#4526), phosphorylated ATR (#2853), phosphorylated P53 (#9286), phosphorylated BRCA1 (#9009), or phosphorylated histone 2A.X (#9718); 2) Lab Vision (Fremont, CA) against Ku70 (#MS-329), Ku80 (#MS-332), and DNA-PK (#MS-370); and 3) Santa Cruz Biotechnology, Inc. (Santa Cruz, CA) against Rad50 (sc-20155), MRE11 (sc-5898), Nibrin (sc-8580), Ku86 (sc56136), ATM (sc-23921), ATR (sc-28901), and PARP1(sc-8007). ATM inhibitor KU55933 was purchased from Tocris (Ellisville, MO) and dissolved in DMSO at 10 mM.

### Flow cytometric analysis and cell sorting

Cells were trypsinized and suspended in PBS plus 1% fetal bovine serum at a density of 1×10^7^ cells/ml. Antibodies against CD44 (# 555478, BD Pharmingen, San Jose, CA), CD24 (#555428, BD Pharmingen) or epithelium marker CD326 (ESA) (#234208, BioLegend, San Diego, CA) were added at dilutions of 1∶10 to 1∶20 as suggested by the manufacturer and incubated on ice for 40 minutes. After washing with PBS containing 1% fetal bovine serum, the labeled cells were first gated to CD326 and then the CD326 positive cells were sorted by CD44 and CD24 markers on a FACSVantage Flow Cytometer/Cell Sorter. The sorted subsets of cells were cultured for 5–6 days.

### Radiation, cell proliferation and clonogenic survival assays

2×10^5^ cells suspended in 2 ml of complete medium in 15 ml plastic tubes were exposed at room temperature to radiation at doses ranging 2 to 8 Gy from a Cesium-137 irradiator. For proliferation assays after exposure to radiation, cells were grown in 96-well plates at initial densities of about 5,000 cells/well in complete culture medium containing 10% FBS. The cell proliferation assay was conducted 5 days after radiation exposure with the CellTiter 96® AQ_ueous_ Non-Radioactive Cell Proliferation Assay Kit (Promega, Madison, WI) according to the manufacturer's instruction. For the clonogenic survival assay, radiated cells were plated at densities of 300 to 3000 cells per well in 6-well plates depending on the radiation dose. After 10 days the cells were stained with 0.05% crystal violet and the colony number enumerated by light microscopy.

### Non-homologous end joining assay (NHEJ)

The NHEJ assay was conducted according to our previous work [14]. Briefly, nuclear extracts were prepared within one hour after radiation with NE-PER® nuclear extraction reagents and 20–25 µg of nuclear protein was mixed with 400 ng of BamH1 and Xho1 digested pBlueKs plasmid DNA in 50 mM Tris-HCL pH 8.0, 40 mM KOAc, 0.5 mM EDTA, 1 mM ATP, 1 mM DTT, 200 µM dNTPs, 100 µg/ml bovine serum albumin, and varying concentrations of magnesium in a total volume of 50 µl. Following incubation at 37°C for 2 hours, the reaction mixture was treated with protease K and extracted with phenol/chloroform. One half of each reaction was separated on a 0.8% agarose gel and stained with Gelstar (Cambrex Corporation, East Rutherford, NJ). The stained gels were scanned and the percentage of end rejoining was calculated by dividing the sum of dimers and multimers by the sum of monomers, dimers, and multimers.

### *In vivo* ligation assay

The luciferase reporter pGL2 control plasmid (Promega) was linearized with Sma 1 and linearization verified by agarose electrophoresis. Cells with or without radiation were transfected with linearized pGL2 and beta-gal plasmids with Lipofectamine 2000. After 36 hour, cells were lysed and luciferase activity was measured and standardized by beta-Galactosidase activity.

### Evaluation of sphere formation

Single cell suspensions of sorted patient breast cancer cells or cell lines were grown in 96-well low cell binding plates at a density of 200 cells in 200 µl of Dulbecco's modified Eagle's medium/F-12 containing 5 mg/mL insulin, 0.5 mg/mL hydrocortisone1x B27 (Invitrogen, Carlsbad, CA), 20 ng/ml epidermal growth factor, and 20 ng/ml basic fibroblast growth factor. After 12 days, the numbers of formed spheres per well were counted by microscopy.

### Western blot analysis

Nuclear proteins were extracted with NE-PER (Pierce Biotechnology, Rockford, IL). Whole cell extracts were prepared either with RIPA buffer (PBS, 1% Nonidet P-40 (NP-40), 0.25% sodium deoxycholate, 0.1% sodium dodecyl sulfate (SDS)) containing 1× protease inhibitor cocktail (Roche Applied Science, Indianapolis, IN) or with Laemmli sample buffer. Protein concentrations were determined by the BCA Protein Assay Kit (Pierce Biotechnology). Both nuclear and whole cell proteins were separated by electrophoresis on SDS-PAGE, transferred to Hybond ™ ECL nitrocellulose membrane (Amersham Biosciences, Piscataway, NJ), and blocked with 5% non-fat milk in 1× TBST (10 mM Tris-HCL, pH 8.0; 150 mM NaCl; 0.05% Tween-20). The blots were then incubated with the indicated antibodies at 4°C overnight, washed, incubated with peroxidase-conjugated secondary antibodies, and signals detected with SuperSignal Dura or Pico Substrate (Pierce Protein Research Products, Rockford, IL).

## Supporting Information

Figure S1 **Flow cytometric analysis of CD44 and CD24.** Cells from the cell lines MCF-7 (A) and MDA-MB-231 (B) and from primary culture of Patient A (C) and Patient B (D) were stained with APC-conjugated CD326 (ESA), FITC-conjugated CD44, and PE-conjugated CD24 and analyzed by flow cytometry as detailed in the Methods. The percentage of CD326 positive cells is shown in the upper right corner of each panel in the left column. The CD326 (ESA) positive cells were then analyzed for CD44 and CD24 (left panel) with the percentage of cells that were CD44^+^/CD24^−^ (i.e. quadrant 4) presented in the Results. The CD326+/CD44^+^/CD24^−or low^ subsets (P6) and control subsets (P5) were sorted according to the gates as shown in each panel of the right column.
(EPS)

Figure S2 **Analysis of DNA ligation activity among subsets of MCF-7, HCC1937, and MDA-MB-231 cell lines.** **A**. *In vivo* analysis of DNA ligation activity among subpopulations of MCF-7, HCC1937, and MDA-MB-231 cell lines. The pGL2-Control plasmid with the SV40 promoter driven luciferase reporter gene was linearized by Stu1 to create blunt ends between promoter and luciferase reporter gene. The linearized plasmid was transfected into subpopulations of MCF-7, HCC193, and MDA-MB-231 cells prior to and post radiation. The luciferase activity was measured 36 hours after transfection. The luciferase activity was standardized by β-gal activity for transfection efficiency and the data presented as relative light units. B. NHEJ analysis of DNA ligation activity between CD44^+^/CD24^−^ and CD44^+^/CD24^+^ subsets. Sorted MDA-MB231 cells were radiated and nuclear protein was extracted. The analysis of NHEJ activity was conducted with plasmid DNA pBlue ks linearized with BamH1 and Xho1 in the absence or presence of varying concentrations of MgCl_2_.
(EPS)

Figure S3 **Western analysis of expression of Ku70, Ku80, PARP1, and DNA-PKcs among the sorted subsets of cells.** A. MDA-MB-231 cells were sorted into CD44^+^/CD24^−^ and CD44^+^/CD24^+^ subsets and HCC1937 cells were sorted into CD44^+^/CD24^low^ and CD44^−^/CD24^high^ subsets. The subpopulations were radiated as indicated and nuclear extracts were prepared as detailed in the Methods and subjected to western blot analysis to detect KU-70/80 and PARP1. B. Western analysis of expression of DNA-PKc in sorted MDA-MB-231 cells. C. Western analysis of DNA-PKc in sorted MCF-7 cell.
(EPS)

Figure S4 **Western analysis of expression of phosphorylated ATR and the Mre11 and Nibrin.** components of the DNA damage recognizing complex. Western analyses were conducted as in Figure 5. Expression of phosphorylated ATR in sorted MDA-MB-231 cells is shown in A and the expression of Mre11 and Nibrin in sorted MDA-MB-231 and MCF-7 cells is shown in B.
(EPS)
